# Recombinant *Tg*HSP70 Immunization Protects against *Toxoplasma gondii* Brain Cyst Formation by Enhancing Inducible Nitric Oxide Expression

**DOI:** 10.3389/fcimb.2017.00142

**Published:** 2017-04-25

**Authors:** Paulo Czarnewski, Ester C. B. Araújo, Mário C. Oliveira, Tiago W. P. Mineo, Neide M. Silva

**Affiliations:** ^1^Laboratory of Immunopathology, Institute of Biomedical Sciences, Federal University of UberlândiaUberlândia, Brazil; ^2^Laboratory of Immunoparasitology, Institute of Biomedical Sciences, Federal University of UberlândiaUberlândia, Brazil

**Keywords:** immunization, r*Tg*HSP70, alum, *Toxoplasma gondii*, toxoplasmosis

## Abstract

*Toxoplasma gondii* is known to cause congenital infection in humans and animals and severe disease in immunocompromised individuals; consequently development of vaccines against the parasite is highly necessary. Under stress conditions, *T. gondii* expresses the highly immunogenic heat shock protein 70 (*Tg*HSP70). Here, we assessed the protective efficacy of r*Tg*HSP70 immunization combined with Alum in oral ME-49 *T. gondii* infection and the mechanisms involved on it. It was observed that immunized mice with r*Tg*HSP70 or r*Tg*HSP70 adsorbed in Alum presented a significantly reduced number of cysts in the brain that was associated with increased iNOS+ cell numbers in the organ, irrespective the use of the adjuvant. Indeed, *ex vivo* experiments showed that peritoneal macrophages pre-stimulated with r*Tg*HSP70 presented increased NO production and enhanced parasite killing, and the protein was able to directly stimulate B cells toward antibody producing profile. In addition, r*Tg*HSP70 immunization leads to high specific antibody titters systemically and a mixed IgG1/IgG2a response, with predominance of IgG1 production. Nonetheless, it was observed that the pretreatment of the parasite with r*Tg*HSP70 immune sera was not able to control *T. gondii* internalization and replication by NIH fibroblast neither peritoneal murine macrophages, nor anti-r*Tg*HSP70 antibodies were able to kill *T. gondii* by complement-mediated lysis, suggesting that these mechanisms are not crucial to resistance. Interestingly, when in combination with Alum, r*Tg*HSP70 immunization was able to reduce inflammation in the brain of infected mice and in parallel anti-r*Tg*HSP70 immune complexes in the serum. In conclusion, immunization with r*Tg*HSP70 induces massive amounts of iNOS expression and reduced brain parasitism, suggesting that iNOS expression and consequently NO production in the brain is a protective mechanism induced by *Tg*HSP70 immunization, therefore r*Tg*HSP70 can be a good candidate for vaccine development against toxoplasmosis.

## Introduction

Because there is no current effective vaccine for humans, prevention of toxoplasmosis transmission seem to be the best method for controlling the disease (Verma and Khanna, 2013). Vaccination is an alternative and more practical way of controlling infectious diseases, including toxoplasmosis (Innes and Vermeulen, 2006; Jongert et al., 2009). Currently, the only commercially available vaccine used to prevent toxoplasmosis is derived from live attenuated *T. gondii* (non-cyst-forming S48strain), Toxovax, and it is used in sheep and goats to avoid abortion (Buxton and Innes, 1995), although the safety of this vaccine for humans and food-producing animals is uncertain. Thus, the recombinant technology to produce antigens to be used as vaccine is an important alternative for immune protection.

To date, several antigenic proteins from *T. gondii* have been extensively characterized, being r*Tg*HSP70 among one of the most antigenic proteins (Xia et al., 2008; Ma et al., 2009). Various recombinant antigens have been used in vaccination protocols to induce protection against toxoplasmosis, 20 μg rSAG1 adsorbed on 0.5 mg Al(OH)_3_ (Petersen et al., 1998), 10 μg rSAG1 or 10 μg rSAG2, or rSAG1 + rSAG2 emulsified in equal amount of Vet L-10 adjuvant injected by intraperitoneal route (Yang et al., 2004), 10 μg rROP1 emulsified in complete Freund's adjuvant (CFA) injected subcutaneously (Sonaimuthu et al., 2016), 10 μg rGRA2 or 10 μg rGRA5 emulsified in CFA administrated subcutaneously (Ching et al., 2016), 10, 20, 30, or 40 μg phosphoglycerate mutase 2 (rTgPGAM 2) intranasally (Wang et al., 2016) only confer partial protection against acute toxoplasmosis. Additionally, there are others studies showing a protective role of recombinant antigens from *T. gondii*, 4 μg rSAG1 or 4 μg rSAG1 plus IL-12 injected subcutaneously or 10 μg rSAG1 plus Heat-labile enterotoxin (LT) instilled into the nostrils, respectively (Letscher-Bru et al., 1998; Bonenfant et al., 2001), 10 μg rGRA4 or 10 μg rROP2 adsorbed in Al(OH)_3_ by intramuscular injection (Martin et al., 2004) or rROP2 + rGRA4 + rSAG1, rROP2 + rROP4 + rGRA4, rROP2 + rROP4 + rSAG1 at concentration of 10 μg of each antigen emulsified with CFA by subcutaneous injection (Dziadek et al., 2012), 20 μg rGRA2 formulated in monophosphoryl lipid A injected subcutaneously (Golkar et al., 2007), rSAG1 + rGRA1 + rMAG1 at concentration of 33 μg of each antigen emulsified in CFA administrated subcutaneously and intraperitoneally (Gatkowska et al., 2008), 10 μg rMIC1, 10 μg rMIC4, or 3,3 μg of each rMIC1+4+6 emulsified in CFA by subcutaneous route (Pinzan et al., 2015), 10 μg of each of the following proteins rCDPK6 or rCDPK6 + rROP18 in Poly(lactide-co-glycolide) (PLG) polymers encapsulated or rCDPK6 or rROP18, and rCDPK6 + rROP18 plus Montanide™ ISA 206 VG injected by subcutaneous route (Zhang et al., 2016) that lead to partial protection against chronic infection or brain cyst formation. However, the mechanisms associated with the protection still remain poorly understood.

*T. gondii* is able to express the *Tg*HSP70 when tachyzoites differentiate into bradyzoites (Weiss et al., 1998); and also during bradyzoite differentiation into tachyzoite, during reactivation of toxoplasmosis (Silva et al., 1998). Immunization studies with *Tg*HSP70 gene-encoding plasmid reduce parasite numbers in the brain and protect mice against *Tg*HSP70-induced anaphylactic reaction (Mohamed et al., 2003; Kikumura et al., 2010). This plasmid-based immunization is dependent on the activation of dendritic cells that lead to Th1 polarization and activation of CD8^+^ cytotoxic T lymphocytes that reduce parasite numbers *in vitro* and *in vivo* (Makino et al., 2011; Chu et al., 2014). On the other hand, immunization of mice with r*Tg*HSP70 emulsified with CFA by intraperitoneal route reduced protective immunity against *T. gondii* infection with RH or Fukaya strain (Mun et al., 1999). Commonly *T. gondii* stage-specific antigens can lead only to stage-limited protection (Alexander et al., 1996). For human toxoplasmosis protection, a good antigen candidate to be use as vaccine should be expressed in both the bradyzoite and tachyzoite life stages.

*T. gondii* is recognized by pattern recognition receptors (PRRs) through which it signals. Glycosylphosphoinositol (GPI) anchors derived from *T. gondii* activates TLR2 and TLR4 (Debierre-Grockiego et al., 2007), and TgHSP70 activates TLR4 (Aosai et al., 2006). Parasite RNA and DNA activate innate immune responses via TLR7 and TLR9 (Andrade et al., 2013) and additionally in rodents the *T. gondii* profilin-like protein is recognized by TLR11 (Plattner et al., 2008) and TLR12 (Koblansky et al., 2013) that are important to IL12 production after *T. gondii* infection. Dendritic cells activated by TLR (Andrade et al., 2013) and inflammatory monocytes (Dunay et al., 2008) produce IL-12 that triggers IFN-γ production by both T helper (Th1) and NK cells (Gazzinelli et al., 1993, 1994); and IFN-γ is essential to control the parasite in acute (Suzuki et al., 1988) and chronic phase (Gazzinelli et al., 1992) of infection. In addition, B cells are key players for the control of *T. gondii* cysts in the brain of wild-type or CD4-defficient mice (Kang et al., 2000; Johnson and Sayles, 2002) and are also important for generation of antibody-mediated immune response in immunized mice that control parasite replication (Sayles et al., 2000). Besides, macrophages represent the main source of nitric oxide (NO), which mediates *T. gondii* parasite killing during acute infection, but its presence also promotes strong intestinal inflammation (Khan et al., 1997). During chronic infection in the brain, inducible nitric oxide synthase-positive (iNOS^+^) cells are found numerously close to *T. gondii* cysts, suggesting a role for controlling chronic infection (Schluter et al., 1999). Also, non-specific iNOS inhibition leads to increased cyst numbers in the brain of chronically infected C57BL/6 mice (Kang K. M. et al., 2004).

Recently, we demonstrated that *Tg*HSP70 is expressed in cysts during chronic infection and that formation of antibody-*Tg*HSP70 immune complexes was associated with better prognosis during infection (Barenco et al., 2014). *Tg*HSP70 is an immunogenic protein that represents a danger signal and is a virulence factor in *T. gondii* infection (Mun et al., 2000a; Dobbin et al., 2002; Ma et al., 2009; Sun et al., 2012). Herein, we tested whether immunization with r*Tg*HSP70 would lead to protection against cerebral cyst formation. We demonstrated that r*Tg*HSP70 stimulation was capable of inducing B cell proliferation and lead to production of high levels of anti-r*Tg*HSP70 antibodies in immunized mice. Surprisingly, immunization with r*Tg*HSP70 in combination with Alum resulted in formation of systemic anti-r*Tg*HSP70 immune complexes, which were associated with reduced histopathology in the brain. Additionally, we observed that r*Tg*HSP70 induced high and persistent NO production in murine peritoneal macrophages, and likewise, we showed that r*Tg*HSP70 immunization enhanced iNOS expression in the brain, which paralleled with lower cysts numbers.

## Results

### Immunization with r*Tg*HSP70 promotes reduction of cerebral parasitism

**Figure 1:**
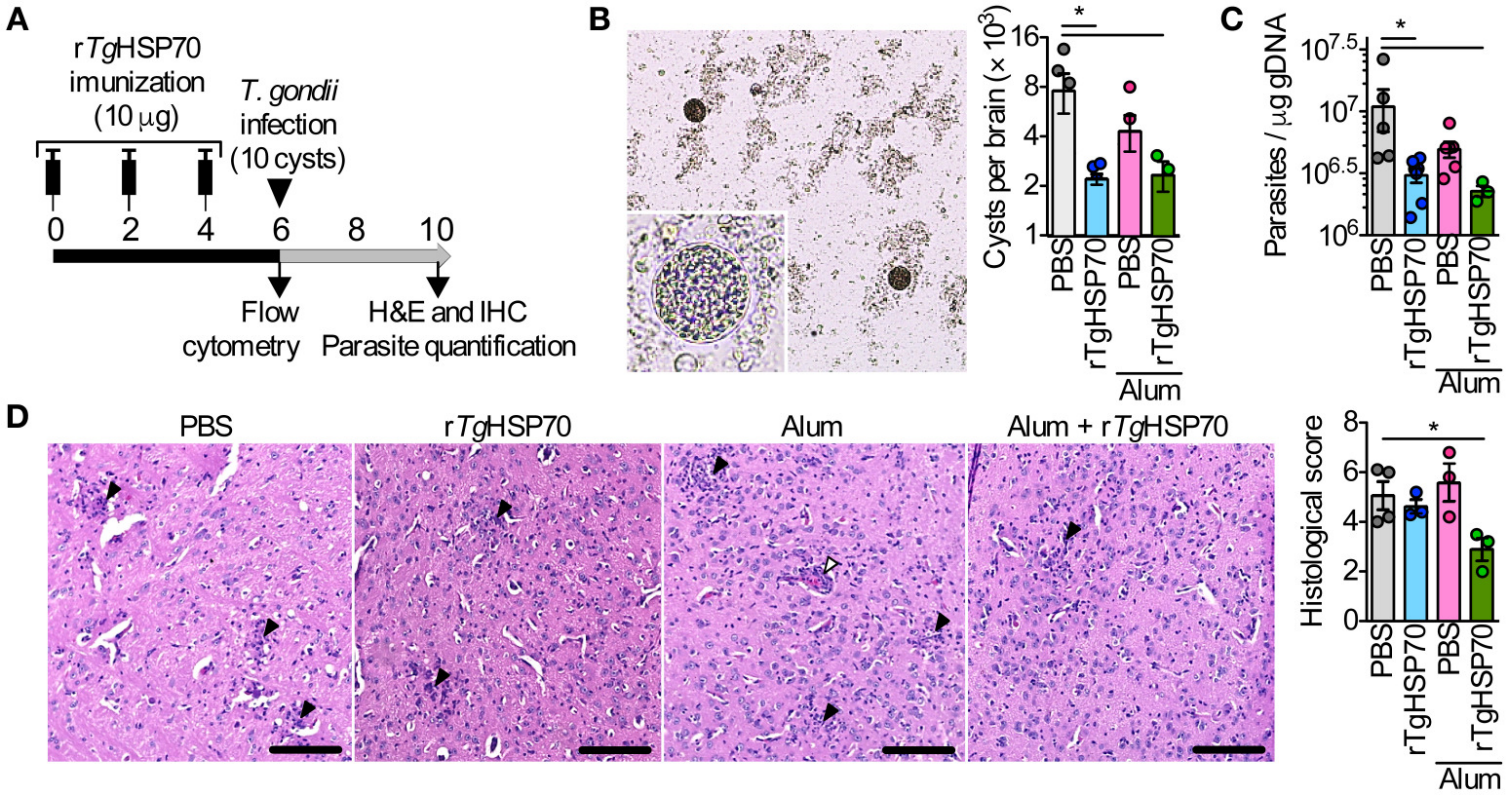
**Immunization with r***Tg***HSP70 reduces brain cyst numbers**. **(A)** C57BL/6 mice were immunized with 10 μg of r*Tg*HSP70 dissolved in PBS or adsorbed in Alum on weeks indicated. After 6 weeks, mice were infected with 10 cysts of ME49 strain of *T. gondii* and were observed for 4 additional weeks. **(B,C)** Parasite quantification by counting cerebral cysts in fresh brain macerates **(B)** and by qPCR for *T. gondii* B1 gene in the brain **(C)** from mice infected. **(D)** Representative micrographs from brain sections and quantification of histological alterations. Inflammatory foci (black arrow heads) and perivascular mononuclear cells (white arrowheads) are pointed. Scale bars represent 100 μm. Data are representative of two independent experiments with five mice per group. ^*^P < 0.05, one-way ANOVA. Error bars indicate mean ± SEM.

Short-term vaccine protocol using r*Tg*HSP70 with ACF, mainly targeting cellular responses, have been described to increase parasite numbers in the brain (Mun et al., 1999, 2000b). Here, we first addressed whether immunization with recombinant r*Tg*HSP70 (Supplementary Figure 1) using Alum adjuvant, has a protective effect against toxoplasmosis. Mice were immunized with r*Tg*HSP70 and/or Alum, challenged with ME49 strain of *T. gondii* at week 6 of immunization and euthanized 4 weeks later (Figure 1A). Mice showed gradual increase of body weight (Supplementary Figure 2A) and no signs of morbidly (data not shown) during immunization. After infection, all groups presented loss of body weight at 9 days after infection with increased morbidity scores, after which mice presented slight gradual weight loss and sustained morbidity score (Supplementary Figures 2B,C). At week 4 of infection, cyst numbers in the brain were evaluated microscopically and parasitism by qPCR. It was observed that mice immunized with r*Tg*HSP70, regardless of vehicle used, presented less cyst numbers in the brain compared with mice injected with PBS alone (Figure 1B) as well as lower parasite B1 copies (Figure 1C). Regarding inflammatory changes, it was observed that mice inoculated with PBS, Alum, or r*Tg*HSP70 alone showed inflammatory changes in the brain characterized by infiltration of inflammatory cells in the meninges, perivascular cuffs, glial nodules, and inflammatory cells throughout the parenchyma. The lesions were also observed in the brain of mice immunized with r*Tg*HSP70 adsorbed with Alum, but in a less extent (Figure 1D). These data suggest that immunization with r*Tg*HSP70 promotes reduction of parasite numbers and, when in combination with Alum, also decrease inflammation in the brain of infected mice.

### Immunization with r*Tg*HSP70 induces high antibody titers, activates preferentially B lymphocytes but does not alter the serum cytokine profile

**Figure 2:**
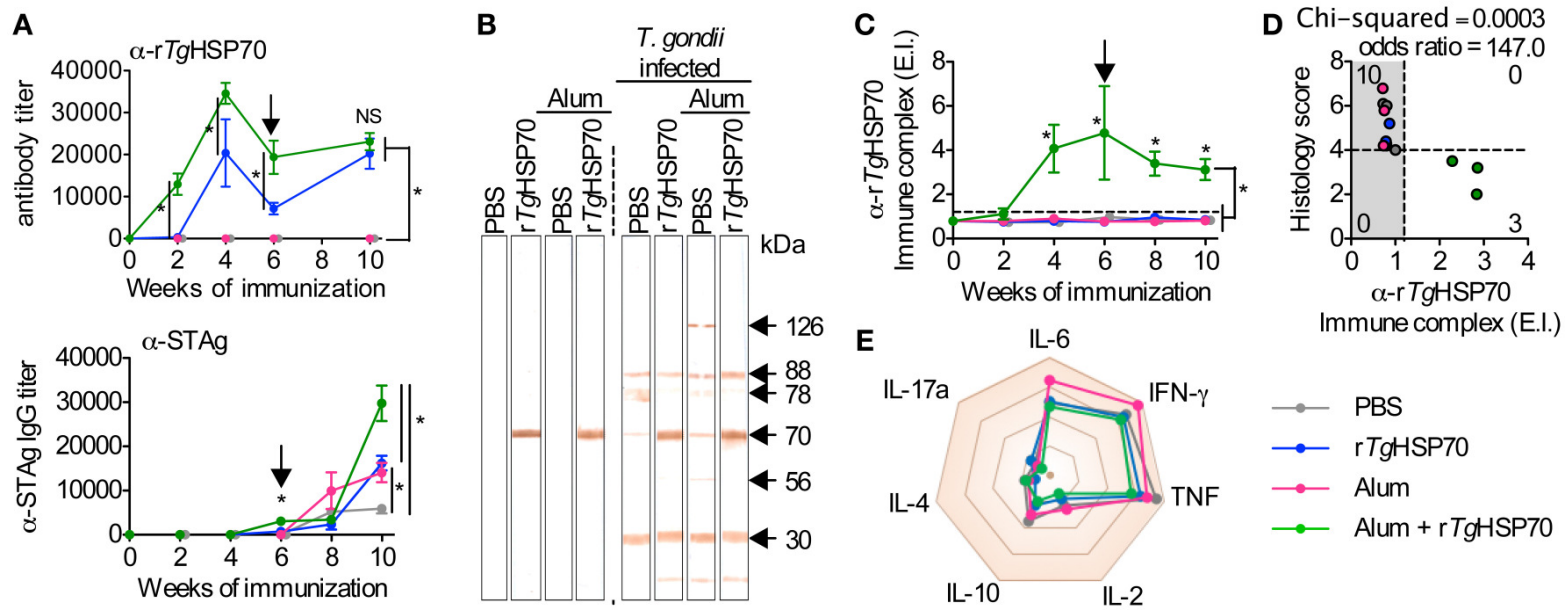
**r***Tg***HSP70 immunization induces strong anti- r***Tg***HSP70 antibody response**. **(A)** ELISA for quantification of total anti-r*Tg*HSP70 and anti-STAg IgG titers from C57BL/6 mice during immunization (week 0–6) and infection period (week 6–10). Arrow indicates when mice were infected. **(B)** STAg proteins were separated by SDS-PAGE and transferred to PVDF membrane. Immunoblotting against-STAg was performed using sera from the each immunization group prior (week 6) and after infection (week 10). **(C)** ELISA for quantification of anti-r*Tg*HSP70 ICs in the serum. IC values above ELISA index threshold (dashed line) indicate the presence of anti-r*Tg*HSP70 in serum. **(D)** Association plot between anti-r*Tg*HSP70 IC and histological score (from Figure 1D) at week 10. Dashed lines represent ELISA index threshold and histological score threshold. **(E)** Radar plot summarizing serum cytokine profile displayed is Sup Figure 4. E.I., ELISA index. Data are representative of two independent experiments with five mice per group. ^*^P < 0.05, two-way ANOVA test **(A,C)**. Chi-squared and odds ratio test were used for association between categorical data **(D)**. Error bars indicate mean ± SEM.

To evaluate the development of humoral response, we measured antibody levels by ELISA. We observed that mice immunized with r*Tg*HSP70 presented specific antibody titers compared with non-immunized groups, and this phenotype was even more significantly distinguished in mice receiving *rTg*HSP70 combined with Alum (Figure 2A). Mice immunized with r*Tg*HSP70 also presented higher levels of IgG1 antibodies, and this phenotype was even higher in animals receiving the protein combined with Alum (Supplementary Figure 3). Moreover, these animals also showed 2–4 times higher titers of specific anti-STAg antibodies than animals injected with PBS alone, especially at week 4 after infection corresponding week 10 of immunization (Figure 2A). Indeed, immunoblotting against STAg showed that sera from immunized mice strongly react with r*Tg*HSP70 (Figure 2B). After infection, all immunized groups recognized protein bands of 30, 70, 78, and 88 kDa, although the animals injected with Alum also recognized 56 and 126 kDa proteins (Figure 2B). To date, several antigenic proteins from *T. gondii* soluble tachyzoite antigen have been extensively characterized, being *Tg*HSP70 one of them (Xia et al., 2008; Ma et al., 2009). Next, we investigated whether immunization could induce circulating r*Tg*HSP70 specific immune complexes (ICs) formation, since we previously showed that BALB/c mice present specific ICs in the sera during chronic infection (Barenco et al., 2014). In the present study, it was observed that mice receiving r*Tg*HSP70 in combination with Alum presented specific ICs formation (Figure 2C) and in parallel reduced inflammation in the brain (Figures 1D, 2D). These results indicate that immunization with r*Tg*HSP70 is able to induce highly specific antibody titers and, when used in combination with Alum, allows formation of circulating anti-r*Tg*HSP70 ICs.

**Figure 3:**
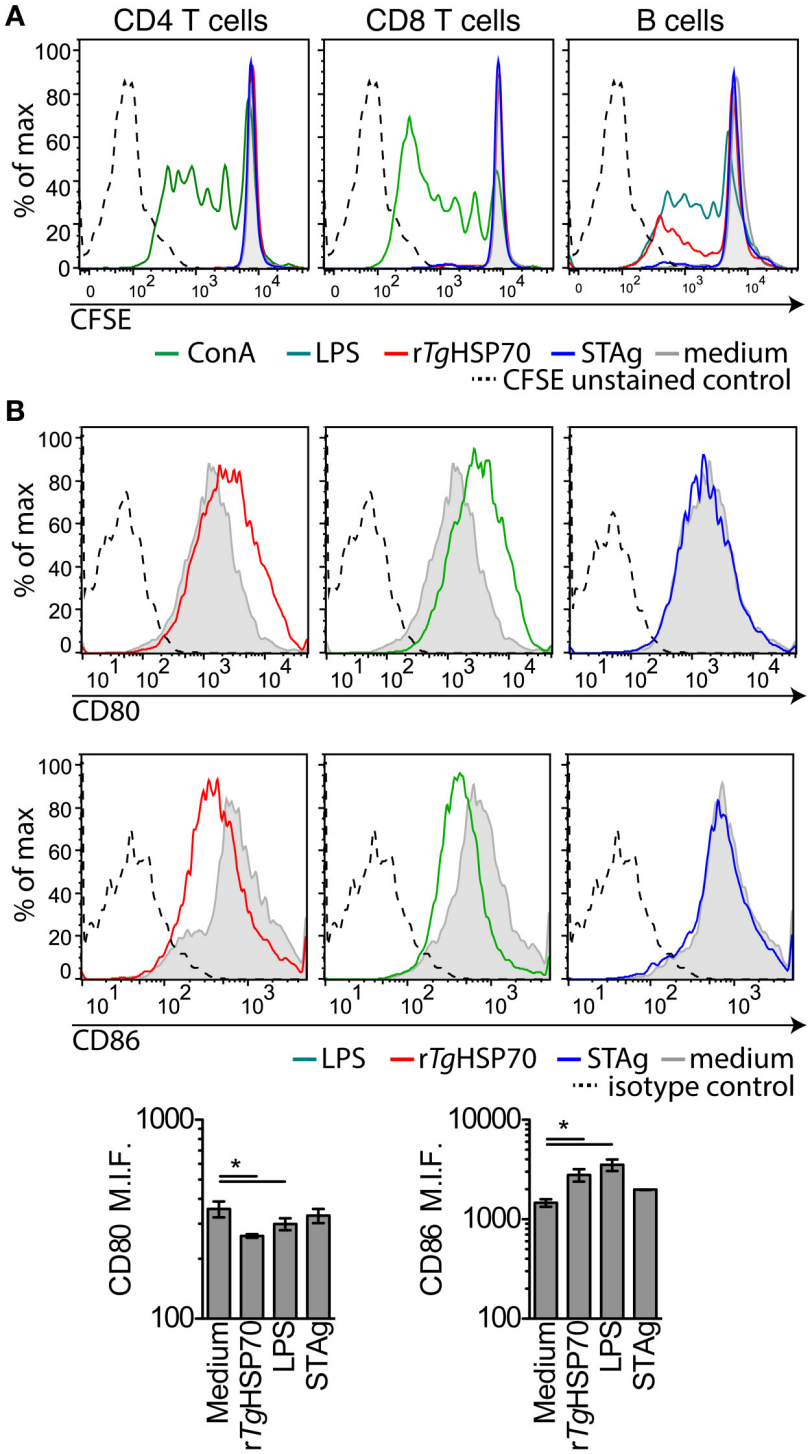
**r***Tg***HSP70 induce B cell proliferation and enhance CD86 surface expression**. Spleen cells from PBS-immunized group were harvested, marked with CFSE and stimulated with r*Tg*HSP70 (10 μg/mL), ConA (2.5 μg/mL), LPS (1 μg/mL), STAg (10 μg/mL), or medium for 72 h. **(A)** Proliferation of B (CD19+), CD4+, and CD8+ lymphocyte populations after incubation with each stimuli, individually. **(B)** B cells were evaluated for expression of CD80 and CD86 surface molecules by flow cytometry. Barplots represent the levels of median fluorescence intensity for CD80 and CD86. Data are representative of two independent experiments with five mice per group. ^*^P < 0.05, one-way ANOVA. Error bars indicate mean ± SEM.

Due to the observed protective effects of r*Tg*HSP70 immunization, we investigated serum cytokines levels in mice before and after immunization and/or infection. It was observed that r*Tg*HSP70 immunization did not alter the production of IL-2, IL-4, IL-6, IL-10, IL-17a, IFN-γ, nor TNF, although infection with *T. gondii* promotes the production of inflammatory cytokines IL-6, IFN-γ, and TNF in all groups of mice (Supplementary Figure 4). These results suggest that immunization with r*Tg*HSP70, regardless of adjuvant used, was not able to modify the cytokine profile in infected mice (Figure 2E). To understand the protective phenotype observed in r*Tg*HSP70-immunized mice, spleen cells were collected at week 2 after the last immunization. Spleen cells were incubated with r*Tg*HSP70 and stained with CFSE for proliferation assay. We observed that *in vitro* r*Tg*HSP70 stimulation was able to induce preferentially B cell proliferation (Figure 3A). T and B cell proliferation phenotype was similar in all groups of immunization (data not shown). Additionally, we found that r*Tg*HSP70-stimulated B cells presented higher CD86 co-stimulatory surface marker, and lower expression of CD80 (Figure 3B) consistent with antibody-producing B cell activation profile (Suvas et al., 2002). The CD86 expression in B cells stimulated with r*Tg*HSP70 was similar in all immunized groups of mice. Thus, these results suggest that r*Tg*HSP70 induces B cell activation by enhancing CD86 surface expression.

### Anti-r*Tg*HSP70 antibodies do not exert function directly on the parasite

**Figure 4:**
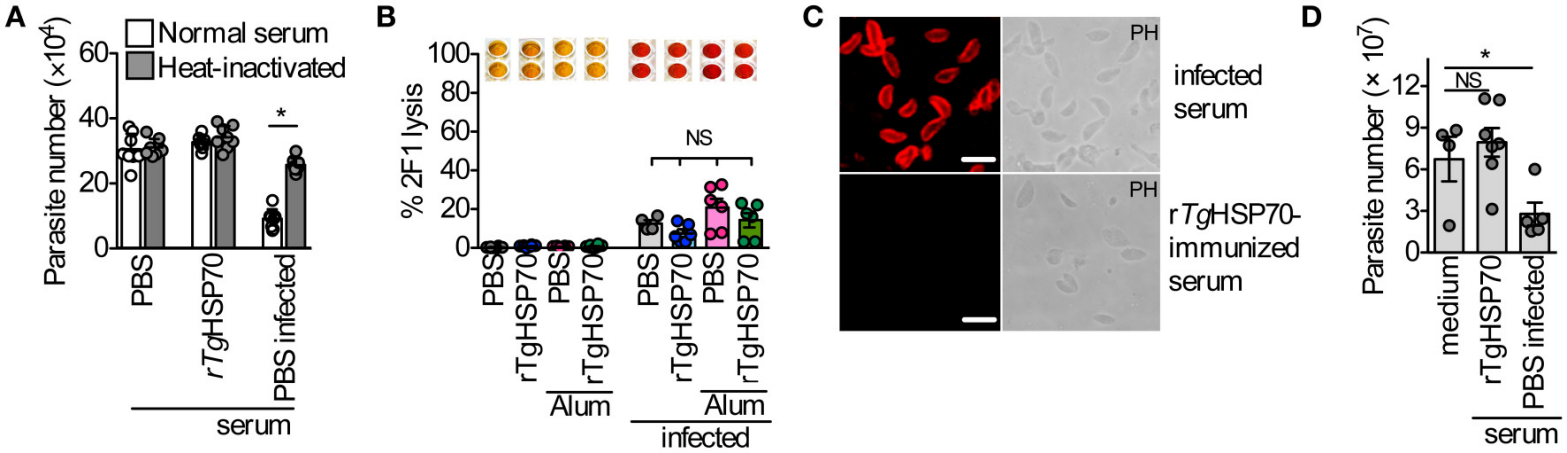
**Anti- r***Tg***HSP70 antibodies do not mediate inhibition and complement-mediated lysis by direct binding to parasite. (A)** 2F1 RH tachyzoites were suspended in medium containing 5% heat-inactivated, or not inactivated, serum from uninfected control (PBS group), uninfected r*Tg*HSP70-immunized or 30 days-infected mice. These parasites were used to infect NIH fibroblasts and parasite numbers was accessed after 24 h. **(B)** 2F1 RH tachyzoites were used for testing complement-mediated lysis using sera from all groups of immunization prior and after infection. Percentage of parasite lysis is shown. Wells above the graphic illustrate the chromogenic reaction quantified by this assay where red color indicates parasite lysis. **(C)** Immunofluorescence using sera from 30 days-infected (PBS group) or r*Tg*HSP70-immunized mice in non-permeabilized *T. gondii* RH tachyzoites. Scale bar represents 10 μm. **(D)** 2F1 RH tachyzoites were incubated with heat-inactivated sera from uninfected control (PBS group) or uninfected r*Tg*HSP70-immunized mice and used to infect C57BL/6 peritoneal macrophages. Parasite numbers were evaluated after 24 h of infection. Data are representative of two independent experiments. ^*^P < 0.05, unpaired two-tailed *t*-test. Error bars indicate mean ± SEM.

There are several possible mechanisms by which antibodies could function protectively in vaccinated mice, including: (1) direct blocking of tachyzoite entry into host cells; (2) replication by phagocytic cells; (3) lysis of extracellular tachyzoites in a complement-dependent manner. In order to verify whether anti-r*Tg*HSP70 antibodies are involved in the control of *T. gondii* directly, we analyzed the involvement of complement parasite lysis by immune serum and the effect of specific antibodies in the parasite replication in non-hematopoietic and hematopoietic cells. It was observed that sera from r*Tg*HSP70-immunized mice did not inhibit replication of RH 2F1 parasites in NIH fibroblasts, as observed with sera from infected mice (Figure 4A). Furthermore, we also checked whether these antibodies might promote complement-mediated parasite lysis. We observed that sera from immunized mice were not able to lyse RH 2F1 tachyzoites compared with sera from infected animals (Figure 4B). It was also observed by immunofluorescence that sera from r*Tg*HSP70-immunized mice were not able to bind to *T. gondii* surface antigens (Figure 4C).

Additionally, it was analyzed the ability of heat-inactivated sera from r*Tg*HSP70 immunized mice to control the proliferation of internalized parasite by C57BL/6 murine macrophages. It was observed that sera from PBS-immunized chronically infected mice were able to control the intracellular parasite, which was not observed with sera from r*Tg*HSP70-immunized mice (Figure 4D). Taken together, these data suggest that anti-r*Tg*HSP70 antibodies do not bind to the parasite surface, and thus neither control parasite numbers by neutralization nor promote complement-mediated lysis.

### Nitric oxide production and *in vitro T. gondii* control by r*Tg*HSP70-stimulated C57BL/6 mice peritoneal macrophages

**Figure 5:**
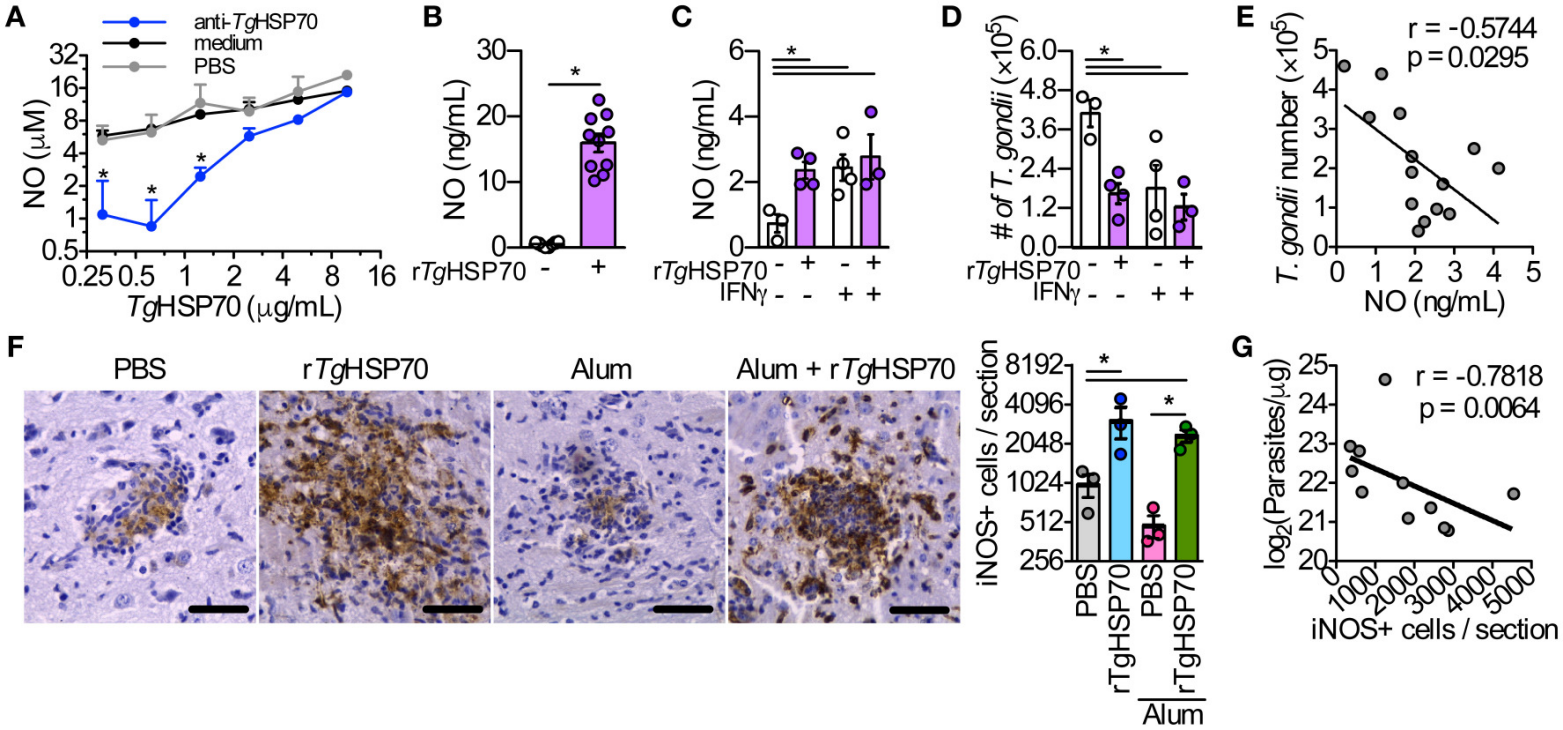
**The stimulus with r***Tg***HSP70 is able to control the parasite ***in vitro*** and r***Tg***HSP70 immunization enhances iNOS expression and reduces parasite load in the brain**. **(A)** RAW264.7 macrophages were incubated with medium containing 2.5% mouse serum from r*Tg*HSP70-immunized group and sera from mice injected only with PBS (control group) mixed with different r*Tg*HSP70 concentrations (10–0.31 μg/mL, 2-fold dilution) for end-point NO quantification by Griess method. **(B)** NO production by peritoneal macrophages after 24 h r*Tg*HSP70 (1 μg/mL) stimulation. **(C–E)** Peritoneal macrophages were incubated with r*Tg*HSP70 (1 μg/mL) for 24 h, washed with medium and subsequently infected with 2F1 RH tachyzoites with/without IFN-γ supplementation (1.3 ng/mL). Readouts were performed 24 h after infection. **(C)** NO production by r*Tg*HSP70 pre-stimulated peritoneal macrophages after infection period supplemented with or without IFN-γ. **(D)** Quantification of parasite numbers by r*Tg*HSP70 pre-stimulated peritoneal macrophages after infection period supplemented with or without IFN-γ. **(E)** Correlation between NO concentration and *T. gondii* numbers from r*Tg*HSP70 pre-stimulated peritoneal macrophages infection period supplemented with or without IFN-γ shown in **(C,D)**. **(F)** Immunohistochemistry for detection and quantification of iNOS+ cells in the brain of infected mice from all immunization groups. Scale bars represent 40 μm. **(G)** Correlation between the numbers of iNOS+ cells with the number of parasites in the brain of infected mice (log-transformed data from Figure 1C). Data are representative of two independent experiments. ^*^P < 0.05, two-way ANOVA (A), unpaired two-tailed *t*-test **(B)**, one-way ANOVA **(C,D,F)** and Pearson correlation **(E,G)**. Error bars indicate mean ± SEM.

Others have shown that *Tg*HSP70 is able to directly induce NO production in macrophages via TLR2, MyD88, and IRAK4 (Mun et al., 2005). We observed that murine RAW264.7 macrophages presented a dose-response NO production upon stimulation with different r*Tg*HSP70 concentrations, which could be slightly reduced with anti-r*Tg*HSP70 antibodies treatment, but not with sera from PBS immunized group (Figure 5A). Similarly, peritoneal macrophages from C57BL/6 mice stimulated with r*Tg*HSP70 also presented enhanced NO production (Figure 5B). To show that NO production by macrophages is depended on r*Tg*HSP70 integrity, we incubated cells with r*Tg*HSP70 or with heat-denatured r*Tg*HSP70. It was observed that macrophages treated with denatured r*Tg*HSP70 did not produce NO (Supplementary Figure 5). Thus, r*Tg*HSP70-induced NO production was not due to endotoxin contamination and depends on protein integrity.

We asked whether NO production could be sustained for a longer period without r*Tg*HSP70 stimulation and whether this sustained production could be enhance parasite killing. Thus, peritoneal macrophages were first stimulated with r*Tg*HSP70 for 24 h, followed by washing and then infected with *T. gondii* RH 2F1 strain in presence or not of IFN-γ for additional 24 h. Interestingly, macrophages pre-stimulated with r*Tg*HSP70 still were able to produce NO, although in lesser extent than 24 h-stimulated macrophages, but at similar levels compared with IFN-γ-stimulated macrophages (Figure 5C). Pre-stimulation with r*Tg*HSP70 was able to decrease 60% of the parasite proliferation, similar to IFN-γ stimulated macrophages (Figure 5D). Thus, r*Tg*HSP70 pre-stimulation is able to control *T. gondii* proliferation by murine peritoneal macrophages probably through NO production (Figure 5E), in agreement with previous literature showing that elevated NO levels present a Toxoplasmacidal role (Schluter et al., 1999; Kang K. M. et al., 2004).

### r*Tg*HSP70 immunization increases the numbers of iNOS+ cells in the brain, which is correlated with lower parasite numbers

Since it was observed that r*Tg*HSP70 induces NO production by resident peritoneal macrophages of mice and is able to diminish the parasite proliferation *in vitro*, we quantified the iNOS+ cells in the brain. Strikingly, it was verified that PBS-immunized or Alum-immunized mice examined on week 4 after infection presented significantly lower number of iNOS+ cells in the brain when compared with animals immunized with r*Tg*HSP70 alone or r*Tg*HSP70 adsorbed in Alum (Figure 5F). A comparative analysis of iNOS expression and parasite load was done, showing that higher iNOS expression was correlated with lower parasite load in the brain (Figure 5G). Taken together, these results suggest that immunization with r*Tg*HSP70, regardless of the adjuvant Alum, is able to increase the number of iNOS+ cells in the brain, which is highly correlated to diminished parasite load observed in the organ.

## Discussion

It was previously shown that DNA vaccination with *T. gondii* HSP70 has a protective effect by limiting the parasite load in organs of infected mice (Mohamed et al., 2003; Makino et al., 2011). In addition, immunization with 100 μg r*Tg*HSP70 prolonged the survival rate of mice challenged with RH strain, and this effect was more effective when r*Tg*HSP70 was used with the adjuvant ginseng stem-and-leaf saponins (Zhuo et al., 2016). In the present investigation, we were interested in know the effect of the vaccination with r*Tg*HSP70 or r*Tg*HSP70 emulsified with Alum in oral ME-49 *T. gondii* infection and the mechanisms involved in the protection. It was observed that the r*Tg*HSP70 immunization decreased significantly the tissue parasitism in the brain, however there was no difference among animals vaccinated with r*Tg*HSP70 alone and r*Tg*HSP70 plus Alum. Of note is that Zhuo et al. got a significant protection in r*Tg*HSP70 immunization using ten times the recombinant protein (Zhuo et al., 2016) compared with our protocol, but in that case they used to challenge animals, tachyzoites of RH, a type I strain, that is highly virulent in mice and we used the ME-49 a type II strain. Type II and III strains are less virulent; however they predominate and really establish chronic infections in animals and humans (Sibley and Boothroyd, 1992; Howe and Sibley, 1995), induce cysts formation that can be used by the natural, oral route to infect mice. Since 1970s, efforts have been made to development of effective adjuvant, and Alum is one of them and it is licensed for human clinical application (reviewed in Azmi et al., 2014). In accordance with our study, it was recently demonstrated that immunization of BALB/c mice with toxofilin or toxofilin plus Alum also decreased the brain cyst numbers in animals challenged with PRU strain, however there was no difference between animals immunized with toxofilin alone or toxofilin associated with Alum (Song et al., 2017). Interestingly, it was observed less severe inflammatory changes in the brain of mice immunized with r*Tg*HSP70 plus Alum.

The immunization with r*Tg*HSP70 induced high specific antibody levels production mainly on week 4 that decline on week 6, however the challenge with ME-49 induced crescent antibody levels. Additionally, r*Tg*HSP70 immunization induced a mixed of IgG1 and IgG2a isotypes, with higher IgG1 levels than IgG2a, in accordance with higher IgG1 antibody levels and Th2-type immune response expected in vaccination protocols using Alum (Grun and Maurer, 1989). In accordance with our study, immunization of NMRI mice with Alum adsorbed rSAG-1 induced higher specific IgG1 than IgG2a levels, which promoted increased survival in mice challenged with RH strain (Petersen et al., 1998).

We have previously shown that chronically infected susceptible C57BL/6 mice were not able to form immune complexes (ICs) against r*Tg*HSP70 as observed in resistant BALB/c, and this was associated with higher disease severity in C57BL/6 mice (Barenco et al., 2014). In the present study, the r*Tg*HSP70-specific ICs were observed systemically only in mice immunized with r*Tg*HSP70 plus Alum. Interestingly, only mice immunized with r*Tg*HSP70 adsorbed in Alum presented ICs formation and in parallel with milder cerebral inflammation, however both immunized groups of mice presented similar parasitism, suggesting that other mechanisms further ICs formation was controlling the *T. gondii* proliferation in the brain. Others have shown that ICs suppress inflammasome activation (Janczy et al., 2014), and can induce inflammatory macrophages, to produce high IL-10 levels (Ambarus et al., 2012). Although r*Tg*HSP70-specific IC levels could be associated with reduced brain inflammation in mice immunized with r*Tg*HSP70 adsorbed in Alum, the underlying mechanism by which this immunization protocol could be reducing tissue inflammation still needs to be explored.

Since it was observed that immunized mice presented low parasite numbers in the brain and elevated anti-r*Tg*HSP70 IgG in the serum, we thought that high anti-r*Tg*HSP70 antibodies titers could be contributing to control the parasite. In the present study, it was observed that the pretreatment of the parasite with r*Tg*HSP70 immune sera was not able to control *T. gondii* internalization and replication by NIH fibroblast as sera from infected mice, neither by peritoneal murine macrophages. In accordance with our experiments, the antibodies recognizing other surface rhoptry, dense granule and micronema molecules had no effect on MDBK cell invasion (Grimwood and Smith, 1996). Related to hematopoietic cells, other study have shown that pre-treatment of RH tachyzoites with heat-inactivated mouse immune serum confer macrophages the ability to inhibit or kill the organisms once they were intracellular (Anderson et al., 1976). In addition, we demonstrated that anti-r*Tg*HSP70 antibodies are not able to kill *T. gondii* by complement-mediated lysis. Taking into consideration that the *Tg*HSP70 is a cytoplasmic protein (Weiss et al., 1998), we postulated that the effectors mechanisms are not mediated by direct antibody-parasite interaction that could avoid entry and/or replication of the parasite inside cells or by tachyzoite extracellular antibody-and-complement-dependent lysis. Thus, with our experimental procedures we did not get to identify the role of anti-r*Tg*HSP70 antibodies in controlling the parasite numbers in the brain of immunized and infected mice.

As *T. gondii* infection is characterized by a pro-inflammatory response (Gazzinelli et al., 1994), and we observed that r*Tg*HSP70 immunization was able to diminish parasite load, we measured the systemic cytokine production under immunization. The r*Tg*HSP70 immunization irrespective the use of the adjuvant did not alter systemic IL-2, IL-4, IL-6, IL-10, IL-17a, IFN-γ, and TNF production. Interestingly, the immunization with *Tg*HSP70 gene induced Th1 polarization locally at the draining lymph nodes with high mRNA IFN-γ expression on days 3 and 5 after the last plasmid inoculation (Makino et al., 2011). Additionally, immunization with 100 μg r*Tg*HSP70 by subcutaneous route increased IL-4 and IFN-γ mRNA expression by spleen cells of immunized mice (Zhuo et al., 2016) 1 week after the last immunization. The different cytokine profile observed in our study could be due to antigen formulation used, the time after immunization of cytokine measurement, r*Tg*HSP70 doses, via of vaccination used and also that in our study we analyzed the cytokine levels systemically and in the other studies it was measured the mRNA expression and not protein levels.

It was previously shown that when *Tg*HSP70 plasmids are used to vaccinate mice, CD8^+^ and CD4^+^ cytotoxic T lymphocytes are induced and mediate resistance to *T. gondii* infection (Chu et al., 2014). Additionally, percentage of CD4^+^ and CD8^+^ T lymphocytes was higher 1 week after immunization with 100 μg r*Tg*HSP70 (Zhuo et al., 2016). It is known that CD4+ and CD8+ T cells, are recruited to the brain and produce IL-2, IL-10, TNF, and IFN-γ in *T. gondii*-infected mice (Schlüter et al., 1997), being IFN-γ and TNF important activator of NO production (Langermans et al., 1992). Despite, the important protective role of TCD4+ and TCD8 + in immunity against toxoplasmosis (Gazzinelli et al., 1992), *in vitro* we showed by CFSE analysis that r*Tg*HSP70 stimulation of spleen cells obtained from animals on day 15 after the last immunization was able to preferentially induce proliferation of B cells. Also, we observed similar B cells proliferation levels when cells were stimulated *in vitro* with r*Tg*HSP70 or LPS. Accordingly, it was previously shown that LPS activates B cells proliferation through TLR4 (Ogata et al., 2000). Interestingly, r*Tg*HSP70 binds to TLR4 (Aosai et al., 2006), however with our experimental procedure is not clear if the r*Tg*HSP70 activates B cell proliferation through TLR4. Additionally, proliferative B cells presented an important CD86 up-regulation. In parallel, r*Tg*HSP70 immunized mice irrespective Alum produced high specific IgG1 and IgG2a levels. Our results are in accordance with Suvas et al. (2002) who demonstrated that cross-linking of CD86 in spleen antigen-activated B cells enhanced the proliferation and production of IgG1 and IgG2a isotypes. In addition, our data of CD86 up-regulation in B cells by r*Tg*HSP70 are in agreement with others, who have shown that murine HSP70 is able to elicit CD86 expression in dendritic cells (DC) (Basu et al., 2000) and that *Tg*HSP70 can induce maturation of murine bone marrow derived (Aosai et al., 2006) and human monocyte-derived (Kang H. K. et al., 2004) DCs via CD80, CD86, and CD40.

Murine macrophages display a *Toxoplasma* microbicidal activity through reactive nitrogen intermediates production when stimulated with IFN-γ and TNF-α (Adams et al., 1990; Sibley et al., 1991; Langermans et al., 1992). As previously shown (Mun et al., 2005) our findings demonstrated that stimulation of murine macrophages with r*Tg*HSP70 was able to induce NO production at similar levels to IFN-γ stimulus, and that NO production was inversely correlated with *T. gondii* numbers. Finally, we measured iNOS expression in the brain of r*Tg*HSP70 and r*Tg*HSP70 plus Alum-immunized mice challenged with *T. gondii*, since we and others have previously shown that Reactive Nitrogen Intermediates are important components involved in the control of *T. gondii* replication in the Central Nervous System (Scharton-Kersten et al., 1997; Silva et al., 2002). It was observed that r*Tg*HSP70 immunization induced high iNOS expression in cerebral tissue from infected mice irrespective the use of Alum, and iNOS+ cell numbers were correlated with lower parasite load in immunized mice. In accordance, we previously demonstrated that low or no iNOS expression leaded to uncontrolled parasite multiplication in the brain and necrotic encephalitis in *T. gondii*-infected mice (Silva et al., 2009).

In conclusion, our results demonstrated that immunization with r*Tg*HSP70 was able to induce massive amounts of iNOS expression in the brain, which was highly correlated with reduced brain cyst numbers irrespective the Alum used as adjuvant, suggesting that iNOS expression and consequently NO production in the brain is a protective mechanism induced by *Tg*HSP70 immunization (Supplementary Figure 6). Thus, r*Tg*HSP70 can be considered as good candidate for further vaccine development against toxoplasmosis.

## Materials and methods

### Animals

Eight-week-old female C57BL/6 and Swiss Webster mice were maintained at the Federal University of Uberlandia animal facility (Centro de Bioterismo e Experimentação Animal-CBEA/UFU), with 12 h light/dark cycles and free access to food and filtered water. All animal experiments were performed in accordance to Brazilian Government's ethical and were approved by the Animal Experimental Ethics Committee (CEUA) of the Federal University of Uberlandia, under protocol no. 023/11. All procedures including housing and welfare were carried out in accordance with the recommendations in the Guiding Principles for Biomedical Research Involving Animals of the International Council for Laboratory Animal Science (ICLAS), countersigned by the Conselho Nacional de Controle de Experimentação Animal (CONCEA; Brazilian National Consul for the Control of Animal Experimentation) in its E-book (http://www.mct.gov.br/upd_blob/0238/238271.pdf). The Federal University of Uberlandia animal facility (Centro de Bioterismo e Experimentação Animal—CBEA/UFU) is accredited by the CONCEA (CIAEP: 01.0105.2014) and Comissão Técnica Nacional de Biossegurança (CTNBio, Brazilian National Commission on Biosecurity; CQB: 163/02). It was used five mice per experimental group and sample size was calculated using the “resource equation” method (Charan and Kantharia, 2013). All efforts were made to minimize animal suffering and the numbers of mice required for each experiment.

### Cell lines and parasite strains

RAW247.6 and NIH fibroblast cell lines were cultured in 25 cm^2^ flasks in RPMI 1640 medium supplemented with heat-inactivated fetal bovine serum (FBS) (both from Cultilab, Campinas,SP, Brasil) and antibiotics (100 U/mL penicillin and 100 μg/mL streptomycin, Sigma-Aldrich, St. Louis, USA) and maintained in incubator at 37°C and 5% CO2. Transgenic *T. gondii* 2F1 strain (β-gal clone) RH strain parasites (Dobrowolski and Sibley, 1996) was a gift from Dr. Vern Carruthers, Medicine School of Michigan University (USA) and was maintained in NIH fibroblasts by passage every 2 days, cultured in RPMI 2% FBS. *T. gondii* ME49 strain was maintained in Swiss Webster mice infected at least 1 month beforehand. For infection of experimental C57BL/6 mice, ME49 cysts were harvested from Swiss brain, quantified and adjusted to 10 cysts in 0.2 mL.

### Tachyzoite soluble antigen (STAg) and recombinant r*Tg*HSP70 preparation

For STAg preparation, tachyzoites of the RH strain obtained from the peritoneal exudates from BALB/c mice previously infected by intraperitoneal (i.p.) were washed in phosphate-buffered saline (PBS) and centrifuged at 70 × g. The supernatant containing tachyzoites was then pelleted (720 × g, 5 min at 4°C), suspended in PBS supplemented with protease inhibitors, sonicated and centrifuged at 10,000 × g, 10 min at 4°C, as previously described (Gazzinelli et al., 1991).

The recombinant rTgHSP70 was obtained as previously described (Barenco et al., 2014). Protein purity was checked by SDS-PAGE (Supplementary Figure 1) and submitted for endotoxin removal using polimixin B resin (Sigma Chemical Co., St Louis, USA). Final rTgHSP70 preparations were absent of detectable endotoxin, as measured by LAL reagent (Lonza, Walkersville, MD USA).

### Experimental immunization and infection

Mice were injected subcutaneously with 10 μg of r*Tg*HSP70 dissolved in 100 μL of PBS or Alum adjuvant (Alhydrogel 2%, Invivogen, San Diego, CA, USA). Experimental design is illustrated in Figure 1A. Control groups were injected with vehicles only. Mice were injected 2 more times at weeks 2 and 4 after first immunization. On week 6, a group of animals was used for spleen *ex vivo* analysis, and the remaining were orally infected with 10 cyst of *T. gondii* ME49 strain. Four weeks later mice were anesthetized by i.p. injection of Ketamine (Syntec Brasil Ltda, SP, Brazil)/Xylazine (Schering-Plow Coopers, SP, Brazil) and blood samples were collected by puncture of the retro-orbital plexus and animals were euthanized by cervical dislocation. The brains were collected for fresh cysts quantification, histological analysis, and parasite quantification by qPCR. Animals were monitored daily for morbidity scores and every other day for weight changes (Bartley et al., 2006) and the blood samples were collected as described above every 2 weeks for antibody and cytokine analysis in serum samples. When mice reached a cumulative score of 5 on any day or a score of 4 for 2 consecutive days they were euthanized as described (Bartley et al., 2006).

### Histological and immunohistochemical analysis

Brain tissue sections fixed in 10% buffered formalin were processed routinely for paraffin embedding and sectioning. Tissue sections were stained with Haematoxilin and Eosin (H&E) for histological assay. The inflammatory score in the brain was done as previously described (Silva et al., 2009). Briefly, perivascular cuffs and inflammatory cells in the meninges as well as total focal or diffuse inflammatory foci were analyzed in a sagittal section. The inflammatory score was represented as arbitrary units: 0–2, mild; 2–4, moderate; 4–6, severe; and above 6, very severe. The histological analyses were done in two histological sections from each mouse using a 40 × objective by two researchers in a blind manner.

To detect iNOS expression by immunohistochemistry, deparaffinized sections were placed in a humidified chamber, and the endogenous peroxidase activity was blocked with 3% hydrogen peroxide. The antigenic unmasking was done in a microwave oven for 7 min and then incubated with PBS plus 0.3% non-fat milk (Nestlé, São Paulo, Brazil). The slides were incubated overnight at 4°C with rabbit anti-iNOS antibody (Santa Cruz Biotechnology, Dallas, Texas, USA) and then with biotin-labeled goat anti-rabbit antibody (Dako, Glostrup, Denmark) for 1 h at 37°C. Next, the sections were incubated with avidin–biotin-peroxidase complex (ABC kit, PK-4000; Vector Laboratories, Burlingame, CA, USA) for 45 min at 37°C. The reaction was developed with 0.03% H_2_O_2_ plus 3,3′-diaminobenzidine tetrahydrochloride (DAB; Sigma). The sections were counterstained with Harris haematoxylin and examined under a light microscope using a 40 × objective.

### Quantification of tissue parasitism

The number of cysts was assessed by counting *T. gondii* cysts in fresh brain samples. For this, one brain hemisphere of each animal was homogenized in 2 mL of PBS by passing through 27G needle. Then, 20 μL of brain homogenate were placed on microscopic slides for cysts quantification by two researchers, in duplicate, in a blind manner. The number of counted cysts was corrected by the concentration in the solution and multiplied by two to obtain the number of cysts in the whole brain.

The quantification of parasites by qPCR was done with genomic DNA (gDNA) extracted using the Trizol method (Life Technologies, Carlshad, CA, USA) following manufacturer's instructions. Absolute quantification was done using B1 gene (FW: 5′-GGAGGACTGGCAACCTGGTGTCG-3′; RV: 5′-TTGTTTCACCCGGACCGTTTAGCAG-3′) primers with 10 ng gDNA using ABI7500 system with SYBR green (Life Technologies). A standard curve made of gDNA extracted from *T. gondii* RH tachyzoites (1 × 10^3^–1 × 10^7^, 10-fold dilutions) was used. Parasite numbers in the samples were calculated by interpolation to the parasite standard curve.

### Quantification of specific anti-r*Tg*HSP70 IgG and immune complexes in the serum samples

Total IgG, IgG1, and IgG2a serum titers were determined by indirect ELISA. Briefly, plates were coated with 2.5 μg/mL r*Tg*HSP70 or STAg in carbonate buffer for 18 h at 4°C. After washes with 0.05% Tween-20 PBS (PBS-T), the plates were blocked with 5% non-fat milk (Neslté) PBS-T (5% PBS-TM) for 1 h at room temperature (RT), and incubated with serial dilutions (6 points, starting at 1:50) of each sample for 1 h at 37°C. After washes, plates were incubated with 1:2000 of anti-IgG or with anti-IgG1 and IgG2a (all from Sigma) for 1 h at 37°C. Plates were washed again and the reaction was developed with enzyme substrate consisting of 0.03% hydrogen peroxide and 1 mg/mL of o-phenylenediamine (OPD, Sigma) and read at 492 nm with VersaMax microplate reader (Molecular Devives). Antibody titers were determined by linear regression (Crowther, 2009).

Immune complexes detection was performed as described (Chaves-Borges et al., 1999; Barenco et al., 2014), using microtiter plates (Kartell SPA, Novigilio, Milan, Italy) coated with anti-r*Tg*HSP70 IgY (10 μg/mL) diluted in carbonate buffer pH 9.6 and incubated at 4°C overnight. After incubation at 37°C for 1 h, plates were washed and incubated with mouse sera diluted 1:40 in 1% PBS-TM. Next, plates were washed and incubated with secondary antibody peroxidase-conjugated anti-mouse IgG (Sigma) diluted 1:1,000. Reaction was developed with OPD (Sigma) and the optical density (OD) was measured at 495 nm by using a VersaMax microplate reader. Immune complex levels were estimated by ELISA index as previously described (Barenco et al., 2014).

### Immunoblotting

STAg antigen was separated by SDS-PAGE and then transferred to PVDF membranes (Millipore Corporation, Billerica, MA, USA), which were blocked with 5% PBS-TM for 1 h and incubated with serum samples diluted 1:100 in 1% PBS-TM overnight. After washes with PBS-T, membranes were incubated with 1:4,000 peroxidase-conjugated anti-mouse IgG (Sigma) in 1% PBS-TM for 2 h, washed again and developed with DAB (Sigma). All steps were performed at RT. Documentation was proceeded on Scanjet G4050 scanner (Hewlett-Packard).

### *Ex vivo* spleen cell proliferation and phenotypic analysis

Spleen cells were isolated from mice 2 weeks after the last immunization, using 70 μm cell strainer and incubated with supplemented RPMI 1640 (10% fetal calf serum, 200 μM glutamine and 50 μM β-Mercaptoethanol). For evaluation of proliferative responses, cells were stained with 5 μM CFSE (Invitrogen, Eugene, Origon, USA) for 10 min, centrifuged and incubated at 2 × 10^5^ cells per well in U-bottom culture plates (Costar 3799, Corning, NY, USA) with 10 μg/mL r*Tg*HSP70 or 2.5 μg/mL ConA or 1 μg/mL LPS or 10 μg/mL STAg in supplemented RPMI 1640 (10% FBS, 200 μM glutamine and 50 μM β-Mercaptoethanol).

Cell phenotyping was performed in cells restimulated *in vitro*. For this purpose, cells were incubated with fluorescent αCD3-BV, αCD4-PECy 7, αCD8-APC, αCD19-APC, αCD80-PE, αCD86-PECy7 (all purchased from BD Biosciences, San Jose, CA, USA), and recorded with FACSCanto II flow cytometer (BD). The results were analyzed using FlowJo software v.10 (TreeStar).

### Cytokine measurement

For serum cytokine quantification, samples were processed using CBA kit following manufacturer's instructions (BD) and the results recorded with FACSCanto-II flow cytometer (BD) and analyzed with FACSDiva software (BD).

### Infection inhibition assays and complement-mediated lysis

Immune serum-mediated *T. gondii* infection inhibition assay was based on a previous publication (Sayles et al., 2000) with modifications. Sera from PBS- and r*Tg*HSP70-immunnized animals and sera from 4 week infected animals were pooled respective to each group. Half of the volume was transferred to another tube for complement inactivation at 56°C for 30 min. Next, sera were diluted (10% [vol/vol]) in culture medium, 0.22-filtered and used to resuspend 2F1 RH tachyzoites followed by 10 min incubation. Parasites (1 × 10^5^) were washed and then used to infect confluent NIH fibroblast culture. Twenty-four hours later, parasite quantification was performed using chlorophenol red–β-D-galactopyranoside (CPRG; Roche, Mannheim, Germany) as described (Dobrowolski and Sibley, 1996).

Evaluation of complement-mediated lysis by immune serum was performed as described (Dando et al., 2001) with modifications. Sera from all immunized groups before (week 6) and 30 days after infection (30 days p.i., week 10) were diluted 1:4 in PBS, incubated at 56°C for 30 min for complement inactivation and added to microtiter plates (75 μL). Meanwhile, ressuspended 2F1 RH tachyzoites (5 × 10^7^ parasites/mL) were mixed v/v with normal mouse serum (pre-diluted v/v in PBS). Next, 50 μL of parasite mixture was added to each well and incubated at 37°C for 15 min and centrifuged at 1,000 × g for 10 min. After that, 50 μL of supernatant was collected and applied to a new plate, in duplicates, for parasite quantification using CPRG (Dobrowolski and Sibley, 1996) without adding lysis buffer.

### Immunofluorescence assay

Formalin-fixed 2F1 RH tachyzoites were placed on microscopic slides and air dried. Slides were incubated at 37°C for 1 h with infected or r*Tg*HSP70-immunized mice sera diluted 1:100 in PBS. After rinsing, slides were incubated at 37°C for 1 h with CF633-conjugated anti-mouse IgG antibody (Sigma) diluted 1:600 in PBS. Finally, slides were rinsed and mounted in glycerin for analysis in LSM 510 Meta Confocal Microscope (Zeiss).

### Nitric oxide assay and infection of macrophages

At first, the influence of r*Tg*HSP70 in nitric oxide (NO) production was measured in RAW247.6 cells, a cell line of BALB/c murine macrophage. Cells were plated (5 × 10^4^ cells/well) and treated with medium containing 2.5% r*Tg*HSP70 immunized-mouse or PBS injected-mouse (control group) sera and different r*Tg*HSP70 concentrations. After 72 h incubation, supernatant was collected for NO quantification by Griess method.

Peritoneal macrophages of C57BL/6 mice were collected and plated (1 × 10^5^ cells/well) in 10% RPMI medium for 24 h at 37°C. The cells were then treated with 1 μg/mL r*Tg*HSP70 and/or 1,3 ng/mL IFN-γ for 24 h at 37°C. The NO production was measured by Griess method, and the previously r*Tg*HSP70 and/or IFN-γ stimulated cells were infected with 2F1-RH tachyzoites of *T. gondii* (3:1) for 24 h at 37°C. The parasitism was quantified using the chlorophenol red–β-D-galactopyranoside assay. The effect of r*Tg*HSP70 denatured by boiling (100°C, 1 h) on NO production analysis was also examined.

In additional experiments, peritoneal macrophages of C57BL/6 mice were plated (1 × 10^5^ cells/well) in 10% RPMI medium for 24 h at 37°C. Cells were infected with 2F1-RH tachyzoites of *T. gondii* (1:1), that were previously incubated by 20 min at 37°C with sera of r*Tg*HSP70 immunized or chronically infected mice. Before the incubation with the parasites all sera were previously heat-inactivated at 56°C for 45 min. Three hours after the infection, cell monolayers were rinsed with fresh RPMI to remove extracellular organisms. Twenty-four hours later, parasite quantification was performed using chlorophenol red–β-D-galactopyranoside.

### Statistical analyses

Statistical analyses were performed using GraphPad Prism 6 (GraphPad Software). Data were expressed as mean ± SEM and were compared using unpaired *t*-test for comparing two groups, and one-way or two-way ANOVA followed by Bonferroni post-test when appropriate. Association between categorical variables (pathology score and IC presence) was done by Chi-square and Odds ratio tests. A threshold of 4 (out of 8) was used to discriminate brain pathological status as “mild inflammation” (scores 1 to 3.9) and “severe inflammation” (scores 4 to 8). Association between continuous variables (NO levels and *T. gondii* numbers) from *in vitro* and *in vivo* experiments was performed using Pearson correlation test. Differences were considered significant when *P* < 0.05.

## Author contributions

NS conceived the idea. NS and PC designed the experiments. TM provided important technical contributions and guidance on experimental design. PC and EA performed *in vivo* immunization experiments. PC and MO performed *in vitro* and *ex vivo* culture experiments. PC and NS analyzed the data. PC, NS, and EA wrote the manuscript. All authors contributed to the manuscript writing.

## Funding

This work was supported by Fundação de Amparo à Pesquisa do Estado de Minas Gerais (FAPEMIG), Conselho Nacional de Pesquisa Científica e Tecnológica (CNPq), and Coordenação de Aperfeiçoamento de Pessoal de Nível Superior (CAPES). NS and TM are research fellows from CNPq.

### Conflict of interest statement

The authors declare that the research was conducted in the absence of any commercial or financial relationships that could be construed as a potential conflict of interest.
