# Supplementary material for: Recombinant TgHSP70 Immunization Protects against Toxoplasma gondii Brain Cyst Formation by Enhancing Inducible Nitric Oxide Expression
Source: Front Cell Infect Microbiol. 2017 Apr 25;7:142. doi: 10.3389/fcimb.2017.00142 (PMC5403831; doi:10.3389/fcimb.2017.00142)
Supplement: Supplementary file 1 [file Presentation1.PDF]

Supplementary Material

Recombinant *Tg*HSP70 immunization protects against *Toxoplasma gondii* brain cyst formation by enhancing inducible nitric oxide expression *in situ*

Paulo Czarnewski, Ester Cristina Borges Araújo, Mário César de Oliveira, Tiago Wilson Patriarca Mineo, Neide Maria Silva*

*** Correspondence:** Dr. Neide Maria Silva: nmsilva@ufu.br

1 Supplementary Figures

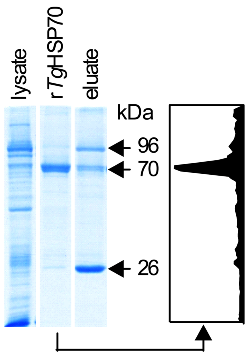

Supplementary Figure 1. Purity of recombinant r*Tg*HSP70 produced in *E. coli*. Recombinant r*Tg*HSP70 (70 kDa) in fusion with glutathione-S-transferase (GST, 26 kDa) was expressed in *Escherichia coli* BL21 DE3 Rosetta upon IPTG supplementation. Proteins from total bacterial lysate, purified *Tg*HSP70 and eluate were submitted to SDS-PAGE. The purity from r*Tg*HSP70 purified fraction is displayed on the right and was above 95%.

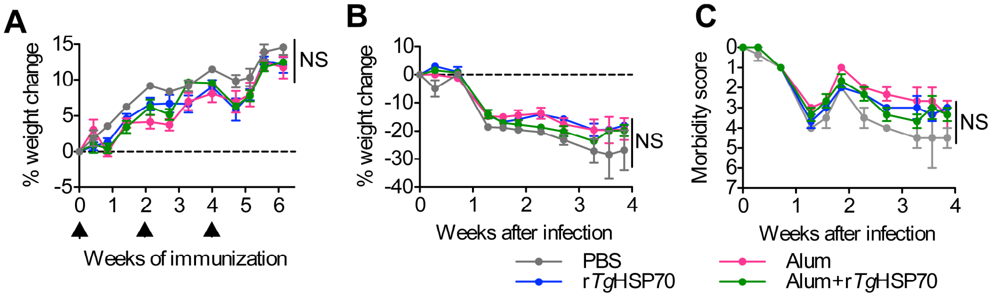

Supplementary Figure 2. r*Tg*HSP70 immunization does not protect mice against body weight loss after *T. gondii* infection. C57BL/6 mice were immunized with 10 µg of r*Tg*HSP70 dissolved in PBS or adsorbed in alum on weeks indicated. After 6 weeks, mice were infected with 10 cysts of ME49 strain of *T. gondii* and accompanied for 4 additional weeks. (**A** to **C**) Mice were evaluated for weight change during immunization period before infection (**A**), and after *T. gondii* ME49 challenge (**B**), when they were also observed for morbidity scores (**C**). Data are representative of two independent experiments. *P<0.05, two-way ANOVA. Error bars indicate mean±SEM.

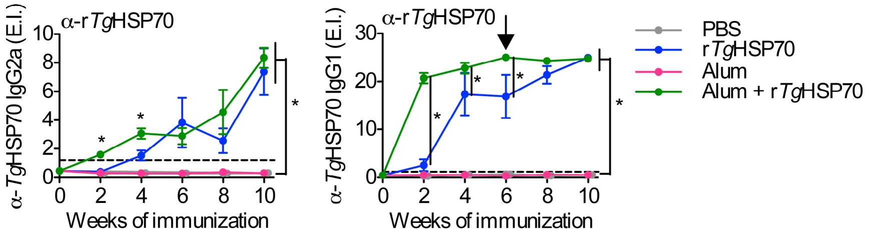

Supplementary Figure 3. Immunization with r*Tg*HSP70 induced higher IgG1 levels compared with IgG2a antibodies. (**A**) ELISA was performed for quantification of anti-r*Tg*HSP70 IgG1 and IgG2a antibodies from C57BL/6 mice during immunization and infection periods. Data are representative of two independent experiments. *P<0.05, two-way ANOVA. Error bars indicate mean±SEM.

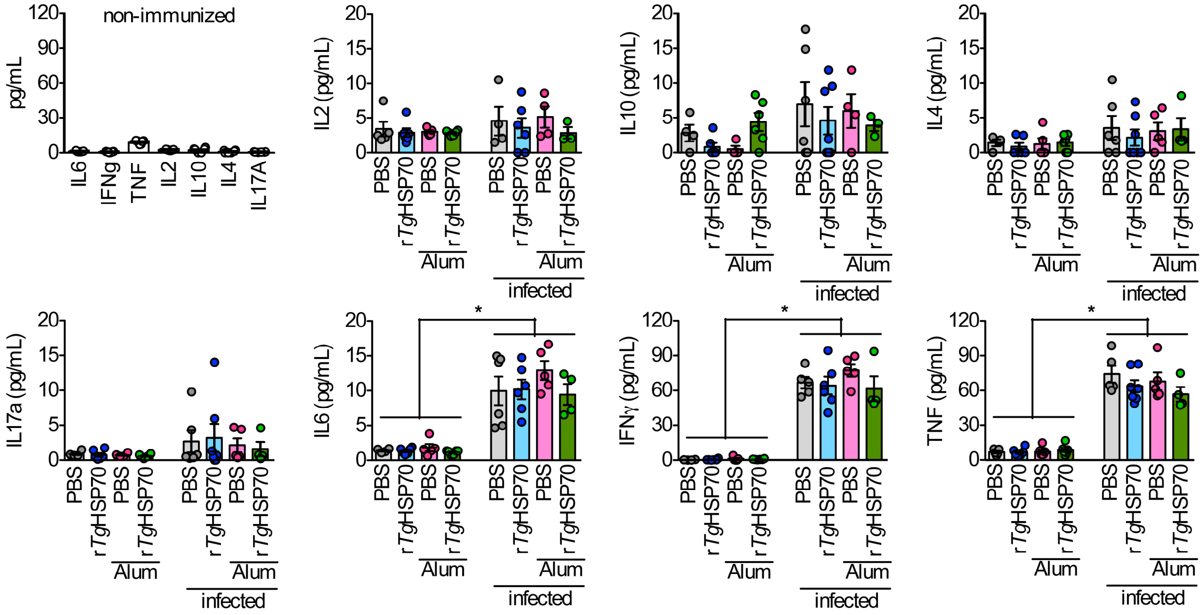

Supplementary Figure 4. *Tg*HSP70 immunization does not change serum cytokine profile. Sera from naïve C57BL/6 mice were evaluated as control for detection of IL2, IL4, IL6, IL10, IL17a, IFNγ and TNF cytokines in the serum samples by CBA. Sera from immunized C57BL/6 mice before (week 6) and after infection (week 10) were analyzed. Data are representative of two independent experiments with at least 3 mice per group. *P<0.05, two-way ANOVA. Error bars indicate mean±SEM.

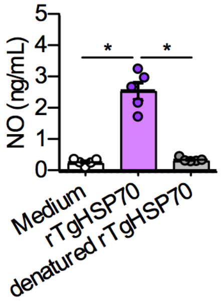

Supplementary Figure 5. Specificity of r*Tg*HSP70 to stimulate NO production by murine peritoneal macrophages. Peritoneal macrophages were incubated during 24 h with r*Tg*HSP70 (1 µg/mL) or heat-denatured r*Tg*HSP70 (1 µg/mL) by boiling (100 °C, 1 hour) and NO production was quantified by Griess method Data are representative of one experiment with 5 replicates. *P<0.05, two-way ANOVA (A). Error bars indicate mean±SEM.

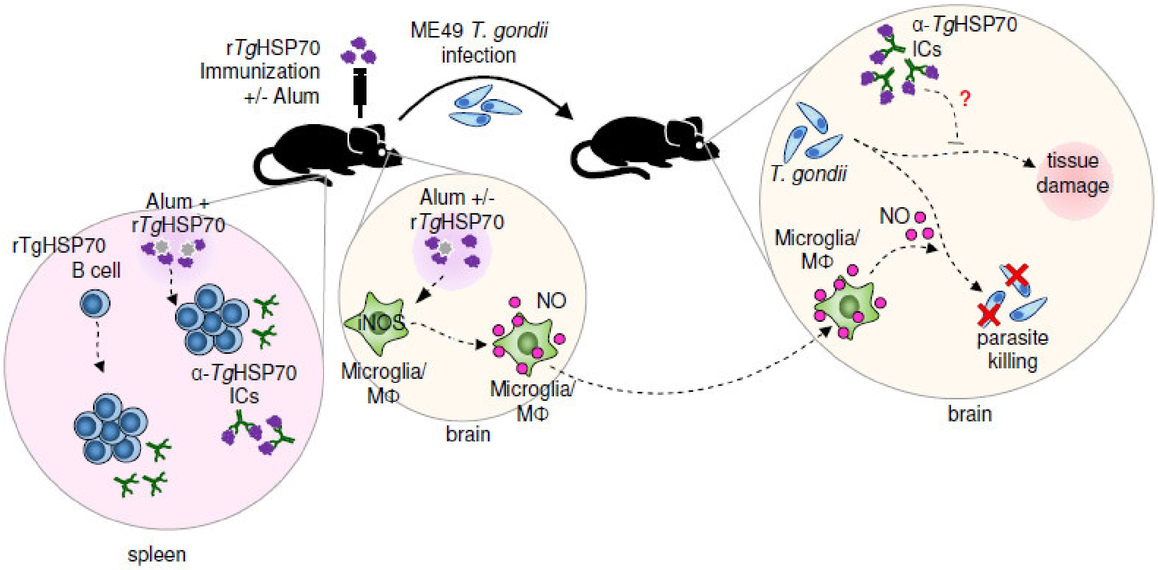

Supplementary Figure 6. Schematic representation of protective mechanism induced by r*Tg*HSP70 immunization of mice. r*Tg*HSP70 immunization irrespective the Alum adjuvant used induces B cell activation and anti r*Tg*HSP70 antibodies production. The specific ICs are formed in r*Tg*HSP70 + Alum immunized mice that could be involved in decreasing tissue damage in the brain, although this mechanism still needs to be further explored. The r*Tg*HSP70 immunization induces iNOS expression and consequently NO production by microglia/macrophage in the brain that could be involved in the decreased parasitism observed in the organ of immunized mice.
